# Comparison of trunk and spine deformity in adolescent idiopathic scoliosis

**DOI:** 10.1186/1748-7161-8-2

**Published:** 2013-01-25

**Authors:** Brandon B Carlson, Douglas C Burton, Marc A Asher

**Affiliations:** 1 Department of Orthopedic Surgery, University of Kansas Medical Center, 3901 Rainbow Blvd, Mail Stop 3017, Kansas City, KS, 66160, USA

**Keywords:** Adolescent idiopathic scoliosis, Computed tomography, Angle of trunk inclination, ATI, Scoliometer

## Abstract

**Background:**

Cobb measurement of standing radiographs is the standard for clinical assessment of coronal spinal deformity. Angle of trunk inclination (ATI) is an accepted clinical measurement of trunk asymmetry, and has variable reported correlations with Cobb angles. Transverse plane spine deformity is most accurately measured using axial computed tomography. Aaro and Dahlbourn’s technique for quantifying apical vertebral rotation with respect to the sagittal plane (RAsag) is commonly reported in the literature. To our knowledge no study has correlated ATI with RAsag. The purpose of this study was to determine the relationship between commonly used measures of trunk and spine deformity.

**Methods:**

Sixteen females that underwent preoperative apical vertebra(e) CT scans were retrospectively studied. Thoracic and thoracolumbar RAsag measurements were date-matched to clinically obtained ATI and Cobb measurements. Two-tailed Pearson correlations were calculated; α = 0.01.

**Results:**

Median patient age was 14.6 years (11–19); BMI 19.4 (16.0-25.5). Curve patterns: Lenke 1 (5); 2 (5); 3 (1); 4 (1); 5 (2): 6 (2). Twenty-six curves (15T; 11TL) with complete, date-matched data points were analyzed. In thoracic curves, ATI correlated with Cobb (*r* = 0.711, *P* < 0.004) and RAsag (*r* = 0.730, *P* <0.003). ATI was inversely correlated with Cobb flexibility (*r* = −0.647, *P* < 0.01). In thoracolumbar curves, ATI correlated with Cobb (*r =* 0.789, *P* < 0.005), and RAsag (*r* = 0.771, *P* < 0.006) but not Cobb flexibility (*r = −*0.452, *P* = 0.190).

**Conclusions:**

Trunk and spine thoracic and thoracolumbar transverse plane deformity are correlated, as are trunk transverse plane and spine coronal plane deformity. Increasing trunk deformity limits thoracic, but not thoracolumbar spine flexibility.

## Background

Adolescent idiopathic scoliosis (AIS) is a three-dimensional deformity of the spinal column and associated rib cage [1]. Coronal and sagittal deformities are typically quantified using 2-dimensional radiographs [2]. Transverse plane vertebral rotation can be assessed using radiographs [2-7], ultrasound [8], and MRI [9], but the gold standard remains axial computed tomography (CT) [10-12].

Trunk deformity is important to both physicians and patients [13]. Many indices of trunk asymmetry exist, each with varying correlations to standard radiographic measurements [14]. The Bunnell Scoliometer is a widely used, non-invasive, clinical method for scoliosis screening [15]. Physicians use a Scoliometer to quantify trunk asymmetry as Angle of Trunk Inclination (ATI) and monitor deformity progression clinically.

Quantifying multiple deformity parameters pre- and post-operatively is useful for understanding AIS disease progression and management effectiveness. Vertebral rotational correction has gained popularity as a means for improving trunk deformity, however, regular assessment using CT is limited by radiation exposures and cost.

The purpose of this study was to quantify the relationship between clinically measured ATI and apical vertebral rotation measured on axial computed tomography. To date, no study has correlated ATI and CT-measured vertebral rotation.

## Methods

This study was approved by the University of Kansas Internal Review Board Human Subjects Committee. It is a retrospective study of prospectively obtained materials for AIS patients treated by a single surgeon (MAA) at a single institution between 1995 and 1998. Inclusion criteria were: 1. Diagnosis of Adolescent Idiopathic Scoliosis 2. Posterior-anterior, lateral and side-bending radiographs 3. CT scans with selective slices at the apical vertebra(e) and sacrum 4. Clinic records documenting ATI measurement(s) 5. All materials must be from the exact same date 6. No previous history of spinal surgery.

Patient curves were defined by Lenke’s classification [16]. Coronal and side-bending Cobb angles were measured by a single investigator (BBC). Vertebral rotation was measured on axial CT scans using Aaro and Dahlborn’s technique [17] by a single investigator (BBC). Apical vertebral rotation angle with respect to the sagittal plane (RAsag) was referenced to S1 rotation to normalize variance incurred by patient positioning, as previously described [18-22]. The treating physician, using the standard Adams bend-test position and a Bunnell Scoliometer, prospectively collected all ATI measurements.

Data were tested for normality using the Kolmogorov-Smirnov test and Levene’s test for equality of variance. Descriptive statistics and differences between curve groups were assessed using the two-tailed Student’s independent *t*-test. Correlations between variables were calculated with the two-tailed Pearson’s product–moment correlation coefficient (*r*). Significance was set at α = 0.01.

## Results

Twenty-two of 68 AIS patients treated during this period were identified with apical CT scans performed preoperatively. Of these, 16 females met all inclusion criteria. Median age was 14.6 years ± 2.6 (11–19) and BMI was 19.4 ± 2.8 (16–26). Curve patterns represented were: Lenke 1A (4); 1B (1); 2A (3); 2B (1); 2C (1); 3C (1); 4C (1); 5C (3); 6C (1). Twenty-six curves (15 T; 11TL) with complete, date-matched data points were analyzed (Table 1). One thoracic and two thoracolumbar curves were excluded because of missing ATI values, leaving 94% of eligible T curves and 85% of eligible TL curves analyzed.

**Table 1:** Descriptive statistics of AIS curves, Mean ± SD (Range)

	**Cobb**	**% Flexibility**	**ATI**	**RAsag**
Thoracic (n = 15)	63° ± 15.2	35% ± 14.9*	18° ± 5.8†	17° ± 7.1
	(29–79)	(11–62)	(6–25)	(5–29)
Thoracolumbar (n = 11)	48° ± 13.9	59% ± 15.8*	10° ± 5.1†	18° ± 10.1
	(34–78)	(34–82)	(4–20)	(5–41)

*,† Two-tailed Student’s independent *t*-test, *p* < 0.01.

Mean Cobb angles were T: 63° ± 15.2 (29–79) and TL: 48° ± 13.9 (34–78). Mean Cobb flexibility was T: 35% ± 14.9 (12–62) and TL: 59% ± 15.8 (34–82). Mean ATI was T: 18° ± 5.8 (6–25) and TL: 10.0° ± 5.1 (4–20). Mean RAsag after normalizing to S1 rotation was T: 17° ± 7.1 (5–29) and TL: 18° ± 10.1 (5–41).

When comparing curve groups, thoracolumbar curves had higher percent flexibility (59.2% vs. 35%; *P* < 0.01) and thoracic curves had larger ATI (18° vs. 10°; *P* < 0.01). No other significant differences between curve groups were observed. In thoracic curves, ATI correlated with Cobb (*r* = 0.711, *P* < 0.004) and RAsag (*r* = 0.730, *P* <0.003). ATI inversely correlated with Cobb flexibility (*r* = −0.647, *P* < 0.01). In thoracolumbar curves, ATI correlated with Cobb (*r =* 0.789, *P* < 0.005,) and RAsag (*r* = 0.771, *P* < 0.006) but not with Cobb flexibility (*r = −*0.452, *P* = 0.190).

## Discussion

Characterizing the relationships between different measures of scoliosis deformity is important to best understand the disease implications and management choices. Cobb angles are known to correlate with many deformity indices [23] and remain the gold standard for monitoring deformity progression. Historically, coronal and sagittal plane deformity correction have been surgical priorities, however, newer instrumentation systems aim to correct deformities in three planes. Recently, emphasis on correcting apical vertebral rotation in the transverse plane has gained popularity as a method for correcting trunk deformity [24].

Computed tomography is the gold standard for quantifying transverse plane apical vertebral rotation, however, its utility is limited by high radiation exposures and cost. Many trunk deformity indices exist and of these, ATI is widely accepted and used clinically. To date, the relationship between trunk deformity indices and apical vertebral rotation is not well described. The present study is the first attempt to quantify CT-measured vertebral rotation (RAsag) with clinically obtained ATI.

Our findings indicate trunk deformity correlates well with spine deformity and vertebral rotation in both thoracic and thoracolumbar curves. We found slightly stronger correlations in thoracolumbar curves as compared to thoracic, which may be due to restrictions imposed by the associated ribcage. Alternatively, this difference may be an artifact of sample size differences despite the result’s significance.

We found a significant negative correlation between ATI and curve flexibility in thoracic curves only. This suggests that as trunk deformity increases, thoracic curves become less flexible. This relationship was not significant in the thoracolumbar region. Our finding is consistent with studies showing thoracic curves are less flexible than thoracolumbar curves in skeletally immature patients [25]. We believe the flexibility mechanisms are different in the thoracic and thoracolumbar curves due to the constraints of the associated rib cage in the thoracic spine and our findings support this theory.

A previous study by Grivas et al. found spine and rib cage deformities may not correlate in younger patients [26]. With our small sample size, we are unable to perform reliable subset statistical correlations based on age groups within the thoracic and thoracolumbar groups. It is possible our correlations will not hold true in younger age groups, however, there are many differences in our study worth noting. All of our subjects have surgical-size curves (T: 63° TL: 48°), whereas their study was based on screening-size curves (T: 22° TL: 17°). They analyzed correlations between arbitrarily created age groups, 7-13 yrs and 14-18 yrs. It is unclear how many of their subjects had progressive curves or the clinical significance of their age groupings. Peak Height Velocity more reliably groups patients for maximal curve progression compared to other maturity scales [27]. Our study only included patients with progressive curves requiring spinal arthrodesis and analyzed the correlation between spine and trunk deformities at the point of maximum progression, prior to surgery. It is unknown whether spine and trunk deformities correlate in patients with progressive idiopathic scoliosis when their curve sizes are small, prior to clinically significant progression. It is possible these spine and trunk deformity correlations will differ between patients with progressive and non-progressive AIS and future studies should attempt to answer this question.

Our findings validate previously assumed relationships between vertebral rotation and trunk asymmetry. This finding establishes ATI as a good surrogate measure of vertebral rotation and may decrease the need for CT scans preoperatively in AIS patients. We believe ATI to be a useful clinical measure of scoliosis progression, vertebral rotation, and thoracic curve stiffness in AIS patients with progressive curves.

Our findings are limited by the retrospective design, small sample size, heterogeneous group of curve types, selection-bias during prospective collection, single-observer radiographic measurements and application in AIS patients with surgical-size curves only. In addition, this study includes both major and compensatory curves, however, all curves were large enough to produce clinically evident trunk asymmetry measureable by a Scoliometer. Inclusion of various curve sizes is important for establishing correlations statistically.

The strengths of this study include date-matched data without missing data points. The senior treating physician used a standardized technique to obtain all ATI measurements prospectively which reduces potential interobserver measurement errors inherent to this measure [28]. Lastly, an investigator not involved in patient care obtained all radiographic measurements.

## Conclusions

Clinically obtained Angle of Trunk Inclination correlates well with coronal Cobb and CT-measured apical vertebral rotation (RAsag) in thoracic and thoracolumbar AIS curves preoperatively. In thoracic curves, ATI negatively correlates with curve flexibility. Clinical use of ATI as a surrogate measure of apical vertebral rotation in un-operated, surgical-size AIS curves may reduce the need for computed tomography, thus reducing radiation exposures and cost.

## Abbreviations

AIS: Adolescent idiopathic scoliosis; ATI: Angle of trunk inclination; T: Thoracic; TL: Thoracolumbar; CT: Computed tomography; RAsag: Rotational angle with respect to the sagittal plane.

## Competing interests

Two of the authors has or had a financial agreement with a commercial company related directly or indirectly to the subject of this research. No financial support was received in direct support of this study. There was no commercial sponsor for this study. All funding for this study was from private research funds.

## Authors’ contributions

MA and DB conceived the study. MA and BC collected and maintained the data. BC performed the statistical analyses. MA, DB and BC were all involved in study design, coordination, manuscript drafting and approval of the final manuscript.
